# Upregulation of GPR109A in Parkinson’s Disease

**DOI:** 10.1371/journal.pone.0109818

**Published:** 2014-10-17

**Authors:** Chandramohan Wakade, Raymond Chong, Eric Bradley, Bobby Thomas, John Morgan

**Affiliations:** 1 Department of Physical Therapy, Georgia Regents University, Augusta, Georgia, United States of America; 2 Department of Pharmacology & Toxicology and Neurology, Georgia Regents University, Augusta, Georgia, United States of America; 3 Department of Neurology, Georgia Regents University, Augusta, Georgia, United States of America; Indiana School of Medicine, United States of America

## Abstract

**Background:**

Anecdotal animal and human studies have implicated the symptomatic and neuroprotective roles of niacin in Parkinson’s disease (PD). Niacin has a high affinity for GPR109A, an anti-inflammatory receptor. Niacin is also thought to be involved in the regulation of circadian rhythm. Here we evaluated the relationships among the receptor, niacin levels and EEG night-sleep in individuals with PD.

**Methods and Findings:**

GPR109A expression (blood and brain), niacin index (NAD-NADP ratio) and cytokine markers (blood) were analyzed. Measures of night-sleep function (EEG) and perceived sleep quality (questionnaire) were assessed. We observed significant up-regulation of GPR109A expression in the blood as well as in the substantia nigra (SN) in the PD group compared to age-matched controls. Confocal microscopy demonstrated co-localization of GPR109A staining with microglia in PD SN. Pro and anti-inflammatory cytokines did not show significant differences between the groups; however IL1-β, IL-4 and IL-7 showed an upward trend in PD. Time to sleep (sleep latency), EEG REM and sleep efficiency were different between PD and age-matched controls. Niacin levels were lower in PD and were associated with increased frequency of experiencing body pain and decreased duration of deep sleep.

**Conclusions:**

The findings of associations among the GPR109A receptor, niacin levels and night-sleep function in individuals with PD are novel. Further studies are needed to understand the pathophysiological mechanisms of action of niacin, GPR109A expression and their associations with night-sleep function. It would be also crucial to study GPR109A expression in neurons, astrocytes, and microglia in PD. A clinical trial to determine the symptomatic and/or neuroprotective effect of niacin supplementation is warranted.

## Introduction

Inflammation is thought to play a central role in Parkinson’s disease (PD) pathology [1]. After the initial report that demonstrated the presence of microglia in the substantia nigra in post mortem samples [2], further research have demonstrated the role of activated microglia and cytokines in clinical and animal studies [3]. In addition, the use of non-aspirin non-steroidal anti-inflammatory drugs was found to reduce the risk of PD [4].

GPR109A (also known as hydroxycarboxylic acid receptor 2 (HCAR2), niacin receptor 1 (NIACR1), HM74a in humans and PUMA-G in mice) is a G protein-coupled anti-inflammatory receptor. It is present in macrophages and neutrophils, at higher levels of expression than other human organs and tissues [5]. Its anti-inflammatory role is well-established in in-vivo and in-vitro studies [6]–[9]. The physiological ligand of GPR109A is beta-hydroxy butyrate (BHB). However, BHB levels are generally not high enough in inflammatory conditions to elicit an anti-inflammatory response. GPR109A has a high affinity for niacin (also known as vitamin B3 or nicotinic acid) which also acts as its agonists and help suppress inflammation [10], [11]. We propose that GPR109A is a crucial part of the chronic inflammation and microglia activation in PD in the substantia nigra and this inflammatory state correlates with GPR109A levels in the blood macrophages. Although macrophages are crucial in inflammation [12]–[14], it remains unknown how they engage and remain engaged with microglia in PD. GPR109A up-regulation both in macrophages and microglia is an indication for anti-inflammatory therapy. Niacin supplementation may reduce the participation of these activated microglia and macrophages in ongoing neuroinflammation.

Niacin is a precursor of nicotinamide adenine dinucleotide (NAD) that is regulated by the oscillating sleep-wake circadian cycle, thereby influencing the oxidative pathways in the mitochondria [15]. Impaired NAD levels may thus be negatively impacted by abnormal sleep function and/or low niacin levels [16]–[19]. As a pharmacologic ligand, Niacin but not nicotinamide, acts through GPR109A. However, the dose of niacin as a vitamin supplement (and precursor of nicotinamide and NAD) is much less than what is needed to affect GPR109A as an anti-inflammatory agent.

It is plausible to visualize the effects of neuroinflammation systemically in PD because the blood brain barrier is compromised. Cytokines such as interferon gamma are known to stimulate GPR109A in murine macrophages, producing a pro-inflammatory cascade of events. GPR109A agonists, in contrast, suppress lipopolysaccharide (LPS)-induced inflammation via nuclear factor-ĸB (NFkB) pathway in the gut. GPR109A is present in a variety of human tissues including the brain. GPR109A plays an anti-inflammatory role in the retinal pigment epithelium [20]. BHB is used by neurons as an alternative energy source and was shown to be protective in mesencephalic neurons against 1-methyl-4-phenylpyridinium (MPP+) toxicity [21]. It should be noted that this effect of BHB is attributed to mitochondrial energy generation and likely to be independent of GPR109A. The role of GPR109A in neurological diseases has never been established. Here we demonstrate for the first time, evidence of elevated GPR109A levels and decreased niacin levels in PD patients compared to age-matched controls and their associations with night-sleep function.

## Methods

### Subjects

Fifty-seven subjects participated in the study approved by the Institution’s review board. Twelve subjects were in the Young control group (6 men, 25±1 years old) and 23 in the age-matched control group (13 men, 69±7 years old). The remaining 22 subjects were individuals diagnosed with idiopathic PD [22] (15 men, 71±8 years old, duration of disease = 10±6 years, H&Y = 2.3±0.7, median = 2).

### Ethics

Participants gave written informed consent under protocols approved by the Institutional Review Boards of the Georgia Regents University. Lab personnel accessed only de-identified data.

### Blood sample collection

Subjects who took any form of over-the-counter anti-inflammatory drugs and/or vitamin B3 supplement were instructed to skip them for at least 48 hours before the morning blood draw. No subject was taking high-dose pain prescription drugs at the time of the study. Approximately 8 ml of whole blood was collected from the subjects in purple-top ethylenediaminetetraacetic acid (EDTA) tubes and kept on ice. Whole blood was spun at 2,000×g for 15 minutes in 15 ml tubes. Plasma was separated and stored at −80°C. White blood cells (WBCs) were then collected and placed into fresh tubes, re-suspended in phosphate buffered saline (PBS) and spun at 300×g for 10 minutes. RBCs were collected and stored at −80°C. Supernatant was suctioned out from WBCs. One ml of ACK Lysing Buffer (Lonza cat # 10-548E) was added to the WBC pellet to lyse any existing RBCs and spun again at 300×g for 10 minutes. Supernatant was discarded from the clean WBC pellet. 200 µl of PBS was used to re-suspend the WBCs and aliquots of 50 µl were then stored at −80°C until further analyses.

### Western blot analysis

Western blotting was performed as detailed by Wakade et al. [23]. WBCs were lysed using Sample Buffer Laemmli 2x Concentrate (Sigma-Aldrich Cat # S3401). Samples were vortexed and heated to 100°C for 10 minutes and centrifuged at 14,000 RPM to collect undissolved material. Protein was measured using RC DC Protein Assay Kit (BioRad Cat # 500-0122) according to the manufacturer’s guidelines. An equal amount of protein was loaded for each sample based on concentration into 10% Mini-Protean TGX Precast Gels (BioRad Cat # 456-1036). Samples were run on the gel for 10 minutes at 80 V for and then 50 minutes at 100 V for the length of the gel. Then samples were transferred to nitrocellulose membrane for 1 hour at 100 V. Blots were then blocked with 5% dry milk solution in TBST for 1 hour. Primary antibody from HM74/GPR109A (Bioworld Technologies Cat # BS2605) was then added in fresh 5% dry milk solution in a 1∶1000 ratio and incubated overnight. Blots were washed three times for 5 minutes each in TBST. HRP-conjugated goat anti-rabbit secondary antibody (Jackson Immuno Research Cat # 111-035-003) was diluted in a 1∶1000 ratio in fresh 5% dry milk TBST solution and incubated for 1 hour at room temperature. Blots were then washed three times with TBST for 5 minutes. HRP was detected using GE Amersham ECL western blotting detection reagent (GE Amersham Cat # RPN2106) on autoradiography film (Denville Scientific Cat # E3012). Primary antibody for b-Actin (Sigma-Aldrich Cat # A5441) was used to confirm loading equality.

### GPR109A analyses

White blood cells (WBCs) from PD patients and controls were used to probe for GPR109A expression. Cell lysates were prepared as described above and protein levels were estimated. GPR109A protein was probed using the antibody from Bioworld Technologies.

### Niacin index

Niacin index is calculated as NAD/NADP X 100. RBCs were lysed as described in the Methods outlined in the following kit manuals. To obtain the niacin index (NAD/NADP), the NAD/NADH quantification kit (Sigma-Aldrich Cat # MAK037) and NADP/NADPH quantitation kit (Sigma-Aldrich Cat # MAK038) were used in tandem. 10 µl of RBC samples were used for each kit according to manufacturer’s guidelines in a 96-well plate with included standards. Samples were filtered using a 10 kda MW cut off spin filter (ABCAM Cat # ab93349) spun at 20,000×g for 30 minutes. All colorimetric detection were accomplished using spectrophotometer absorbance at A = 450 nm. The niacin index and the NAD/NADH ratio were then calculated for each sample.

### Total plasma metabolites by HPLC/MS

Fasting blood samples were collected in the purple-top EDTA tubes from nine PD patients and nine age-matched controls (N = 18). Plasma portions were separated and analyzed for niacin metabolites (nicotinic acid, niacinamide and niacinuric acid). Samples were then sent to NMS Labs with a decoder. Detection level was set to 10 ng/ml for each metabolite; less than 10 ng/ml were not detected. The quantitative analysis for niacin and metabolites was performed at NMS Labs (Willow Grove, PA) using LC-MS/MS, liquid chromatography with tandem mass spectrometer detector. Twenty-five µl of deuterated internal standard was added to 0.20 ml aliquot of serum/plasma samples. Samples were pH-adjusted and extracted by solid phase extraction where samples were eluted with basic methanol, dried, and reconstituted with formic acid in water solution, and transferred to vials for instrumental analysis. Analysis was performed on Waters ACQUITY LC system with an Aquasil C18, 2.1×100 mm, 5.0 micron, part number 77505-102130, or equivalent USP L1 column, and TQ MS/MS detector. Quantitation was achieved by monitoring two transition ions following LC separation with positive-ion electrospray tandem mass spectrometry (LC-MS/MS) for each analyte and standard. Each analytical run was independently calibrated at concentrations of 10, 20, 50, 100, 400 and 500 ng/ml. Two levels of control were run in each analytical batch. This LC-MS/MS method has a LLOQ of 10 ng/ml and current between run % CV of 11.40%, and 6.59% at 20 and 400 ng/ml, respectively for nicotinic acid, % CV of 5.61%, and 6.00% at 20 and 400 ng/ml, respectively for nicotinamide, and % CV of 11.40%, and 6.00% at 20 and 400 ng/ml, respectively for nicotinuric acid.

### Beta-hydroxybutyrate (BHB) analyses

Serum samples were filtered using a 10 kda MW cut-off spin filter(ABCAM Cat # ab93349) spun at 14,000 RPM for 30 minutes. BHB was detected on a 96-well plate according to manufacturer’s guidelines (BHB Assay Kit, ABCAM, cat # ab83390).

### Night-sleep EEG test

A subset of 28 subjects from the blood experiment participated in the overnight EEG sleep study: 12 in the Young group (6 men, 25±1 years old), nine in the Older age-matched control group and (one man, 68±6 years old) and seven in the PD group (six men, 73±5 years old, median H&Y = 2). PD subjects’ disease profiles are summarized in Table 1. The Older group comprised mostly live-in spouses of the PD subjects in order to warrant comparable dietary intake and sleep environment. Worse motor symptoms were strongly associated with rating of disease severity and increased prescription of Carbidopa.

**Table 1:** PD characteristics.

	Mean ± SD	Range
**Age (years)**	73±5	66–80
**Disease duration (years)**	8±7	2–22
**UPDRS total**	29±19	7–55.5
**UPDRS brady**	6±5	0–15.5
**H&Y**	2.4±1.0	1–4
**MMSE**	28±2	23–30
**PDQ total**	12±7	4–25
**RAPID1**	5±4	1–12
**RAPID2**	5±3	1–10
**RAPID3**	21±33	0–90
**Symptoms rating**	8±3	1–10

UPDRS total, sum of section 3 of the Unified PD Rating Scale [62].

UPDRS brady, sum of UPDRS section 3 last five items assessing body bradykinesia [26].

H&Y, Hoehn & Yahr disease rating scale [63].

MMSE, mini-mental status examination [64].

PDQ total, sum of PD Quality of Life questionnaire [65], [66].

RAPID1 (Rapid Assessment of Postural Instability in PD item 1), difficulty in performing activities of daily living (0 = no difficulty; 1 = difficulty) [67], [68].

RAPID2, fear of falling (ranging from 1 = “no fear” to 10 “very fearful”).

RAPID3, number of falls over the last three months (including near falls).

Symptoms rating, self-reported rating of PD symptoms, ranging from 1 to 10: 1 = “very bad day” - symptoms are the worse compared to a typical day; 10 = “very good day” - symptoms are absent or minimal compared to a typical day).

**Figure 1:**
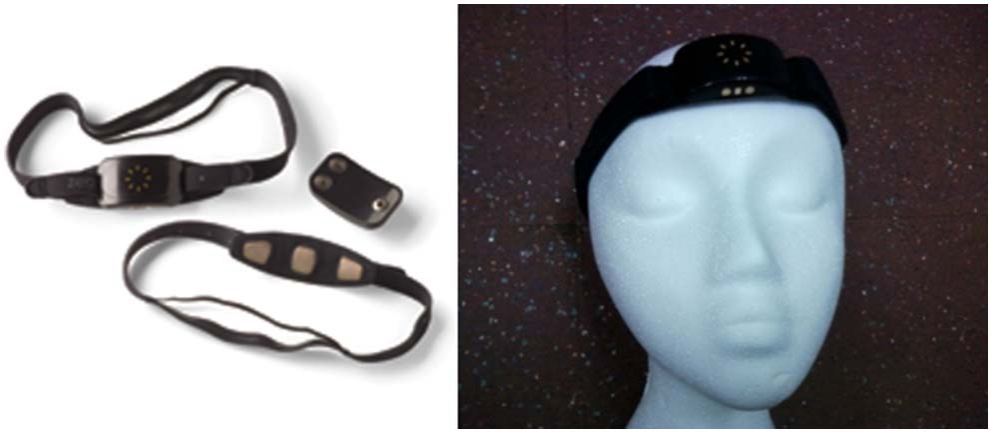
Illustration of the EEG sleep monitor.

The Zeo portable sleep EEG monitor was used to assess the quality of night-sleep. It is a validated, unobtrusive, easy and convenient dry wireless 2-channel EEG system. The monitor is embedded in a headband which contains a lightweight rechargeable battery that lasts 16 hours on a full charge. Subjects wore the headband to sleep on the night after the morning blood draw in which the activities of the day was considered to be routine, i.e. not unduly tired and not preparing or returning from a long trip (Figure 1). The headband has a 12-bit analog-to-digital converter that samples EEG signals at 128 Hz and filters with a second order band-pass of 2–47 Hz. The processed signal was then transmitted via a 2.4 GHz wireless protocol to a receiver station (typically placed on the nightstand). Numeric and graphical results were automatically generated which provided details of the sleep including time to fall asleep, number of times awoken during sleep, and time during dream sleep (REM) and deep sleep. Sleep efficiency (refreshing versus disruptive sleep) were then derived. Sleep efficiency was calculated as the ratio of actual time spent sleeping divided by the total attempted sleep, which is the sum of time taken to fall asleep + actual sleep + time spent awake during night-sleep multiplied by 100. A low percentage value is undesirable because it indicates that the subject either took a long time to fall asleep, awoke in the middle of the night-sleep for a relatively long duration, or both. On the other hand, a high percentage value is desirable as it indicates that the subject fell asleep relatively quickly and did not or rarely woke up in the middle of the night.

Scoring the individual's sleep stages was based on analyzing each 2-second interval using a 2-minute moving average smoothing algorithm. The resulting signal that contained the majority of a particular sleep stage every 30 seconds was reported. The wireless system has been shown to have excellent overall agreement in scoring the various sleep states when compared to the ‘gold standard’ described by Rechtschaffen and Kales in 1968 [24]. The validity of the EEG sleep monitor in scoring the various sleep stages has been compared to a polysomnography in a sleep laboratory. The percent agreement between the two methods was found to range between 74.7% and 95.8%.

### Statistics

A two-sample multivariate t-test (Hotelling's T-square) was used to analyze for differences among the NAD/NADH ratio, NAD/NADP ratio, GPR109A and BHB levels between the Older and PD groups. EEG sleep data were analyzed with 1-way ANOVA followed by Tukey's test for significant main effects. Cohen's *d* was used to determine the effect size for significant results [25]. The Spearman correlation test was also used to determine the associations among the PD subjects' disease profiles, NAD/NADH ratio, NAD/NADP ratio, GPR109A, BHB levels, sleep function and quality of life. Data from the Young group are shown to enable visualization of normative values and were not included in the multivariate or correlation analyses. *p*<0.05 was considered to be statistically significant for all analyses.

## Results

### GPR109A, NAD/NADH and BHB analyses

The correlations among the blood dependent variables (NAD/NADH ratio, GPR109A and BHB) for the combined Older age-matched control and PD data ranged from −0.125 to 0.205 (*p*>0.05). The Group main effect was significant, Wilks's lambda = 0.729, F (3, 42) = 5.20, *p* = 0.0038. Follow-up discriminant function and univariate analyses revealed that GPR109A was the most sensitive variable in differentiating the PD group from the Older age-matched control group (total-sample standardized canonical co-efficient = 0.92), followed by NAD/NADH (–0.75) and BHB (0.36). The 1-tailed univariate analyses corroborated the discriminant function for GPR109A (*p* = 0.009, *d* = 0.68), NAD/NADH (*p* = 0.033, *d* = 0.50) and BHB (*p* = 0.072, *d* = 0.48).

Within the PD group, NAD/NADH levels were positively correlated with BHB levels (r = 0.943, *p* = 0.017).

**Figure 2:**
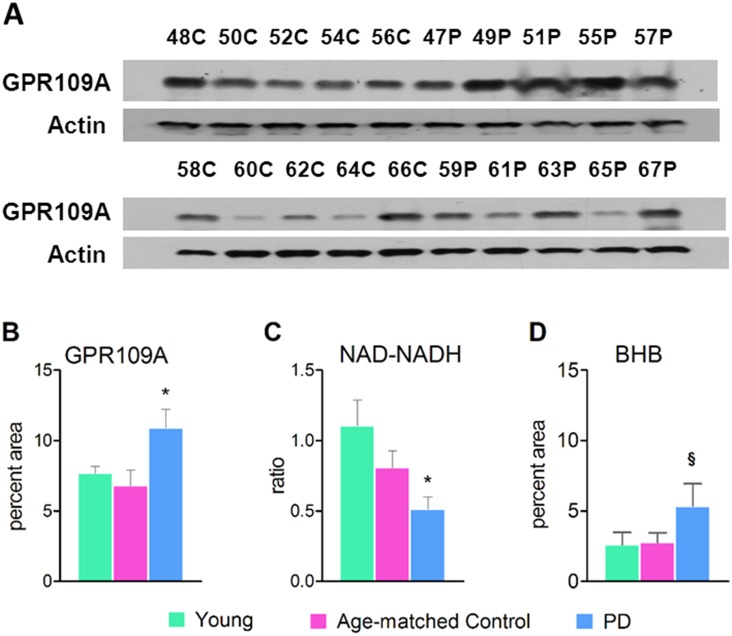
GPR109A expression, NAD/NADH ratio and BHB levels in blood. (A) Representative GPR109A western blots (B) GPR109A densitometry, (C) NAD/NADH ratio and (D) BHB levels. GPR109A expression and NAD/NADH ratio were tested in the WBCs. The BHB levels were tested in the sera. Young, n = 6; Older, n = 23, PD, n = 22. *p = 0.009 between Age-matched control and PD groups. **p = 0.033 between Age-matched control and PD groups.^ §^ *p* = 0.071 between Age-matched control and PD groups.

A representative western blot of GPR109A is shown in figure 2A. The GPR109A was detected at around 45 KD. The densitometry scan shown in figure 2B demonstrates the relative densities of the western blot bands. Twenty out of 22 PD subjects showed up-regulation of GPR109A (*p* = 0.009 between the Older age-match control and PD groups). This may indicate either an active and/or chronic inflammatory state. Whether this is reflective of neuroinflammation is speculative at this point. Beta-actin was used as the housekeeping protein.

The NAD/NADH ratio was significantly reduced in the PD group compared to the Older age-matched controls (*p* = 0.033). Reduced NAD indicates poor mitochondrial function, lethargic Kreb’s cycle and/or increased oxidative state (Figure 2C).

A trend approaching significance in increased levels of BHB in the plasma of PD patients compared to age-matched controls was observed (*p* = 0.071, Figure 2D). BHB is known to fluctuate in a variety of conditions including starvation, diabetes mellitus and other co-morbidities.

The strength of association between the independent variable (Group) and the linear combination of the dependent variables, eta-squared η^2^ was 0.27, indicating that 27% variance in the dependent variables (NAD/NADH ratio, GPR109A and BHB) was attributed to group differences.

### Reduced niacin index (NAD/NADP ratio) in RBCs

**Figure 3:**
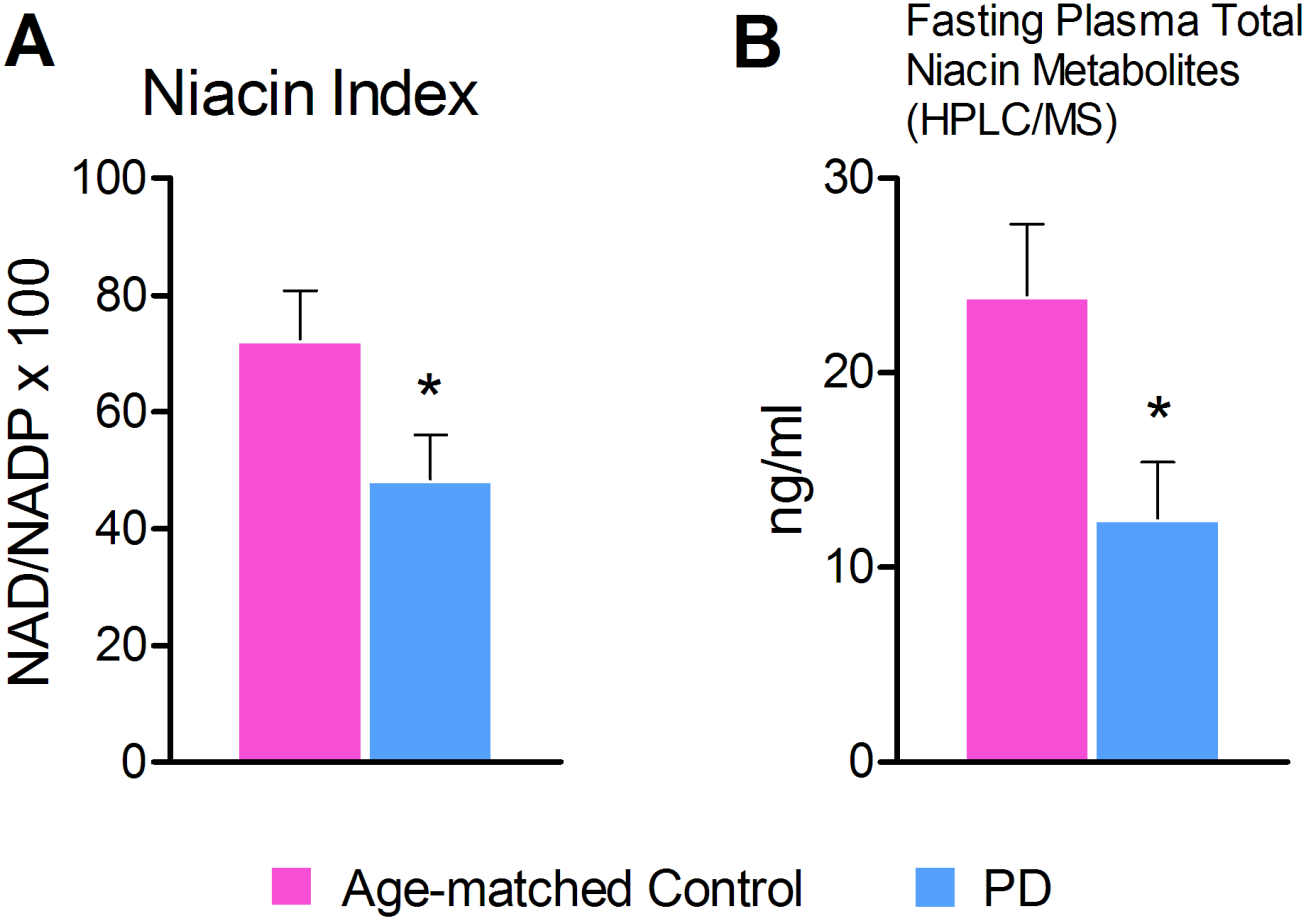
A, Reduced Niacin index (NAD/NADP ratio) in RBCs and B, Total plasma metabolites by HPLC/MS. A. NAD/NADP ratio was significantly reduced in the PD patients compared to age-matched controls (n = 18, p = 0.038). B. Total niacin metabolites from PD patient’s samples were significantly lower than that of their age-matched controls (p = 0.025). This data is in unison with our niacin factor (NAD/NDAP ratio) data.

We observed that NAD/NADP ratio was reduced in the PD group compared to the Older age-matched controls (*p* = 0.038). (Figure 3A).

### Total plasma metabolites by HPLC/MS

Fasting blood samples in the PD group demonstrated lower total niacin metabolites (nicotinic acid, niacinamide and niacinuric acid) than their age-matched controls. (*p* = 0.025). The majority of the samples did not detect nicotinic acid and nicotinuric acid (Figure 3B).

### Increased expression of GPR109A in the substantia nigra of PD brains

**Figure 4:**
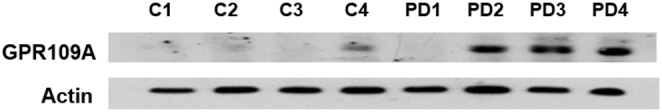
Up-regulation of GPR109A in the substantia nigra of PD patients.

We demonstrate here for the first time the up-regulation of GPR109A in the substantia nigra of PD patients compared to the Older age-matched controls (Figure 4). Three out of the four PD patients showed robust increase in GPR109A expression. Patient PD1 showed an outlier GPR109A level (Grubb's critical Z = 1.48, *p*<0.05). It is possible that the GPR109A was degraded. It is also possible that the patient was taking niacin supplements or anti-inflammatory drugs. The medical records of these individuals are no longer available.

### Co-localization of GPR109A and microglia in PD and control brain

**Figure 5:**
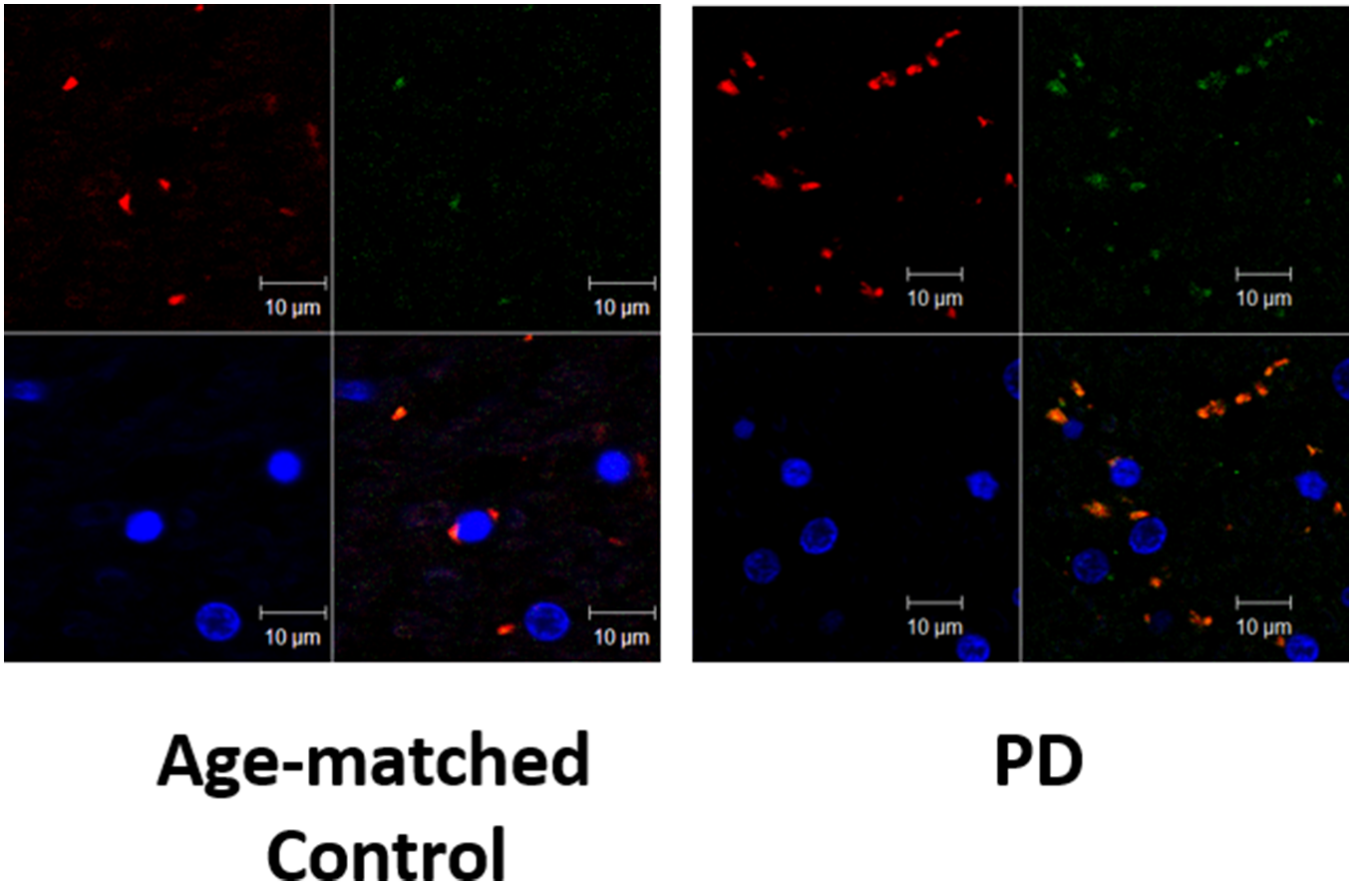
Co-localization of GPR109A and microglia in PD and control brain. Confocal microscopy image of SN of human brain samples showing the glial marker, CD11b (green) co-localized with GPR109A (red). Control sample shows less microglia and GPR109A + cells. Note that all the GPR109+ cells are not co-localized with CD11b maker in control sample. Few neuronal Nuclei (blue) are seen.

Paraffin embedded brain human samples (post-mortem) 20 µm in thickness were stained for GPR109A and microglia. Confocal microscopy images showed that the majority of the microglial marker, CD11b (green) were co-localized with GPR109A (red). Control samples showed less microglia and GPR109A+ cells. Note that in the control samples, not all the GPR109+ cells were co-localized with the CD11b maker. Few neuronal nuclei (blue) are seen (Figure 5).

### Sleep quality

**Figure 6:**
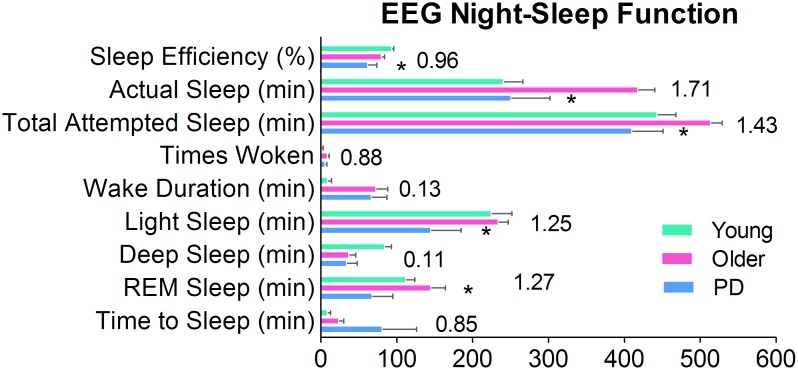
Sleep in PD. Sleep Efficiency, ratio of actual sleep divided by total attempted sleep. Actual Sleep, total duration spent sleeping (not including Wake and Time to Z). Total Attempted Sleep, from time to bed to morning rise. Times Woken, number of times subject awaken during night-sleep. Wake Duration, total duration of time spent awake during night-sleep. Light Sleep, light sleep stage. Deep Sleep, deep sleep stage. REM Sleep, rapid eye movement sleep stage. Time to Sleep, the time it takes to fall asleep (a.k.a. sleep latency). *p<0.05 between the PD and Older groups. (Young group's data are shown in order to visualize normative values.) Numbers are the effect sizes between the Older and PD groups, based on Cohen's *d* using averaged standard deviation [25].

Compared to the Older age-matched group, PD subjects had decreased sleep efficiency. They slept less hours, and spent less than half the amount of time in REM and light sleep (Figure 6). As a percentage of sleep time, the groups were similar. The more severe the level of bradykinesia, the longer it took them to fall asleep (r = −0.76, *p* = 0.028).

Severity of body bradykinesia [26] captured problems with quality of life and sleep function better than overall motor symptom assessment. Worse bradykinesia were associated with less duration of actual night-sleep, increased difficulty with activities of daily living, fear of falling, feeling of depression, higher frequencies of painful muscle cramps, restlessness of legs and higher frequency of falls (Table 2).

**Table 2:** Subject characteristics.

	UPDRS total	UPDRS brady
**UPDRS total**		
**UPDRS brady**	0.893	
**H&Y**	0.982	0.873
**Sleep4**	–0.891	–0.746
**PDQ1**	0.899	0.973
**PDQ3**	0.879	0.805
**PDQ7**	0.786	0.879
**PDQ total**	0.821	0.786
**Times woken**	-	–0.771
**Actual sleep (min)**	-	–0.821
**Light sleep (min)**	-	–0.821
**Carbidopa (mg/day)**	0.973	0.826
**RAPID1**	-	0.901
**RAPID2**	-	0.821
**RAPID3**	-	0.778

Values represent good to excellent coefficient of correlations (*p*<0.05 for all). Empty cells indicate moderate or low correlations (*p*>0.05).

Abbreviations are the same as Table 1.

*Sleep4 (PD Sleep questionnaire item 4), restlessness of legs or arms at night or in the evening causing disruption of sleep [69].

§ PDQ1 (PD Quality of Life questionnaire item 1), difficulty getting around in public.

§ PDQ3, feeling depressed.

§ PDQ7, painful muscle cramps or spasms.

Times woken, number of times woken during EEG night-sleep assessment.

Actual sleep, sum of EEG light sleep, deep sleep and REM sleep durations.

Carbidopa, prescribed with dopamine (as Sinemet) to minimize breakdown of levodopa before it crosses the blood brain barrier.

*High scores indicate less problems.

§ High scores indicate more problems.

### Association between quality of night-sleep and blood analyses

The PD characteristics and sleep correlation analyses are summarized Table 1 and 2 respectively. Higher BHB levels were marginally associated with higher NAD/NADH ratios (*r* = 0.750, *p = *0.066). Lower NAD/NADH ratio and BHB levels were associated with higher frequency of painful muscle cramps in the extremities and higher frequency of painful posturing of the extremities. Conversely, higher NAD/NADH levels were associated with a longer duration of deep sleep while higher levels of BHB were associated with lower frequency of tiredness and sleepiness in the morning (Table 3).

**Table 3:** Associations among PD NAD/NADH ratio, GPR109A, BHB and sleep quality.

	NAD/NADH	GPR109A	BHB
**Sleep11**	0.793	-	0.901
**Sleep12**	0.775	-	0.955
**Sleep14**	-	-	0.927
**EEG deep sleep (min)**	0.786	-0.75	-

Values represent good to excellent coefficient of correlations (p<0.05 for all). Empty cells indicate moderate or low correlations (p>0.05).

*Sleep11, painful muscle cramps in arms or legs while sleeping at night.

*Sleep12, wake up early in the morning with painful posturing of arms or legs.

*Sleep14, feel tired and sleepy after waking in the morning.

*High scores indicate less problems.

## Discussion

### Role of GPR 109A and PD

We are the first to report here the up-regulation of GPR109A expression in the blood and the substantia nigra of PD patients. It is an additional indication of an ongoing inflammatory process in PD, and potentially opens novel avenues of detection and treatment [27], [28]. Up-regulation of GPR109A in the WBCs and microglia (substantia nigra) of PD patients suggests the need for GPR109A agonist therapy such as niacin. It is possible that different dosages and durations of niacin therapy will be required in combatting inflammation via GPR109A and increase NAD levels to boost anti-oxidative mechanisms. Although it remains to be seen whether niacin supplementation or other agonists of GPR109A help ameliorate PD symptoms, we have demonstrated a novel proof of concept which warrants further investigation. A “cross-talk” between peripheral macrophages and CNS microglia may be pivotal in keeping the neuroinflammation ongoing. GPR109A may play an important role in this crosstalk.

### Niacin index (NAD/NADP ratio) and PD

NAD/NADP ratio in erythrocytes is an indirect way to indicate the niacin index in the body. Altered NAD/NADP ratio has been implicated in pellagra and other neurological conditions. These levels are also known (especially NAD) to respond to niacin treatment. There are multiple roles of NAD in cellular metabolism. It’s a critical co-enzyme for three rate limiting steps in the TCA cycle. The role of NAD in the longevity of mitochondria and efficient mitochondrial function through silent information regulator 2 (SIRT2) pathway has been shown [29], [30]. Other studies have also raised the potential therapeutic role of other NAD precursors [31]–[33]. Exogenous application of NAD precursors, such as nicotinic acid mononucleotide, nicotinamide mononucleotide, and nicotinamide riboside protected against axonal degeneration after axotomy [34]. These studies suggest that maintenance of cellular bioenergetic homeostasis and NAD levels are crucial to support the NAD-dependent enzymes, such as enhancing SIRT1 activities, and for protection against excitotoxicity [32]. Altered NAD-NADP ratio (niacin index) has been implicated in ATP depletion and mitochondrial dysfunction, causing aging [35]–[37] and neuronal death in neurodegenerative diseases like PD [32].

### Sleep disorders in PD

NAD levels are shown to couple mitochondrial bioenergetics with light-dark cycle in mice [15]. Lower NAD levels were associated with impaired mitochondrial function in clock mutant mice.

REM sleep, the majority of which takes place in the later part of night-sleep, was reduced by less than half the duration of that in the Older controls. It has been suggested that this phase of night-sleep may be an important non-motor symptom of PD including those in the early stages of the disease [38]–[41]. The disordered REM phase is manifested in the lack of inhibition of the neuromuscular system. However, patients may not always experience daytime sleepiness. It is possible that the night-sleep disruptions are not severe enough [42] and therefore patients may not seek medical advice. REM abnormalities may thus be under-reported [43].

Although it is well-known that many PD patients have abnormal REM sleep patterns, our study also indicates that they do not get enough of it as well. Abnormal REM sleep combined with inadequate deep sleep in subjects with low levels of niacin produced an overall low quality of night-sleep.

### Relationship between decreased niacin levels, body pain and abnormal sleep in PD

Our discovery of the associations between niacin deficiency and frequent pain in the body and decreased sleep efficiency in PD are novel. These associations may be related to loss of nuclear SIRT1 activities, which is known to produce abnormal sleep cycles [44]. Treatment of moderately old mice (first-phase OXPHOS defects) with nicotinamide mononucleotide (NMN) (NAD precursor) increased oxidative phosphorylation activity and other markers of mitochondrial function in the skeletal muscle within one week without altering muscle strength [45]. The cellular mechanisms of how low NAD levels are associated with sleep disorders in the development and progression of PD remains to be determined [46]–[49].

### General discussion: GPR 109A agonists for reduction of inflammation

There are multiple agonists of GPR109A that might be useful as anti-inflammatory agents, including BHB, niacin, and Na butyrate [50]. Alisky et al. [51] reported an account of a man with PD who was initially given 500 mg of niacin daily to treat his high triglyceride level. The treatment appeared to work and a higher dose of 1000 mg was subsequently attempted. Three months later, during a follow-up primary care visit, the patient's family reported an unexpected positive side-effect of the niacin treatment in the form of an increase in his physical functioning. They included the ability to rise from a chair (which he previously was unable to do without assistance) and being able to walk faster (which he previously was very slow to execute due to freezing). These improvements were thought to be attributed to a noticeable decrease in his rigidity and bradykinesia, the classical symptoms of PD. The niacin dosing however, was too high, which may explain the nightmares and skin reactions. A lower dose that can elicit similar symptomatic relief without side effects would be noteworthy [28]. It needs to be further clarified whether the above-mentioned effect of niacin was due to its metabolic effects or its effects on GPR109A or the combination thereof.

Similarly, two case-control studies found that those who consumed a niacin-rich diet had a decreased risk of developing the disease, after correcting for occupational and environmental factors [52]. In addition, the carbidopa medication that PD patients take depletes niacin levels in the body [53]. Thus, niacin depletion may worsen PD prognosis.

The neuroprotective role of niacinamide is documented in MPTP and other models of PD in mice [54], [55]. The role of NAD, decreased apoptosis (by blocking PARP pathway), decreased oxidative stress and inhibition of NOS have been implicated as possible mechanisms involved in neuroprotection by niacinamide [32]. Niacin has been shown to be neuroprotective in animal stroke models [56]. Niacin was thought to be involved in vascular and axonal remodeling of the animals. However, there is no published data that demonstrates the neuroprotection of niacin in any PD animal model. Both niacin and niacinamide are sources of NAD. Niacin but not niacinamide acts as an agonist of GPR109A. Therefore, although both niacin and niacinamide are neuroprotective, their mechanisms appear to be different. Multiple second messenger pathways are associated with GPR109A which are helpful for cell survival. Their pathways and cell types involved in PD pathology are still unknown.

Butyrates (GPR109A agonists) are also known to inhibit inflammation through inhibiting NFκB in Crohn’s disease [57]. Butyrates decrease pro-inflammatory cytokine (TNFα, IL-6 and IL-1β) expression via inhibition of NFκB activation and IκB degradation [58]. Butyrates also inhibit NFκB activation via GPR109A and increases IκB levels in-vitro in intestinal epithelial cell lines [59].

It would be interesting to see if and how up-regulation of GPR109A modulates the homing of microglia in the substantia nigra. GPR109A appears to be a new plausible prognostic marker and a pharmaceutical target in the treatment of PD. Niacin supplementation may not only serve as an anti-inflammatory agent but also replenishes NAD levels which are essential for healthy mitochondrial function as well as dopamine synthesis [60], [61]. The effects of niacin on the NAD/NADH ratio, GPR109A levels and functional recovery in PD patients need to be studied in a clinical trial with tight control on drug intake and dietary habits [28].
